# Supplementary material for: Revealing the Regulatory Mechanism of lncRNA-LMEP on Melanin Deposition Based on High-Throughput Sequencing in Xichuan Chicken Skin
Source: Genes (Basel). 2022 Nov 17;13(11):2143. doi: 10.3390/genes13112143 (PMC9690664; doi:10.3390/genes13112143)
Supplement: Supplementary file 1 [file genes-13-02143-s001.zip › Supplementary Table S1 .pdf]

Table S1. Information of primers sequences

Gene	Primer sequence (5'-3')	Tm (℃)	Product (bp)	Application
F1	GCGGTGGGTGTTCAGCATGG	60	1579	RACE
R1	CATGCACCCACGTGCACTGT			
F2	CAGAGTAGGACGTGGTTCGG	60	1476	
R2	TGTAAATCCTGGTGATAGCC			
3'-F	GAATCGAGACAACTACACAGCACT	55	1600	
5'-R	GACTTGAAGCCGCCACGTACATCT	55	350	
ATP5E	TGAACAGTCTTCCAGCGTGC<br>CAGGTGCACAGAAGTTCGTC	60	249	qRT-PCR
SLMO2	GAAGCCTTCAGGCAATGCAC<br>TCGCACGTTACACAGAGGTC	60	163	
TYR	TTGACAGCATTTTTGAGCGGTG<br>CTGATGGGCTTGCTTGAGGT	60	198	
EDN3	TCAACACCCCAGAGAGGACT<br>GCAAACGTTAAACCTTCTTCTGTC	60	179	
TCONS_00070905	CGGTTAAGGCACAACTGGAA<br>CTTTCCCTGAGGGGCATT	60	176	
TCONS_00072039	AGGGTGACAACAGTGGCATT<br>GCCGCTGTCATTAACGTAAC	60	176	
TCONS_00054154	AGCTGAGAACTTGGCAGACC<br>CTGTAACGTGCCAGAAACGC	60	249	
Gallus_gallus_newGene_1037	TGAGCCCTGTGAGACATCTG<br>TCTTGCTTGTGCCTGCATAC	60	206	
Gallus_gallus_newGene_720	GGCCACATCCATGCTATCTT<br>GGCTTCTCAAAGTGCCTCAC	60	212	
β-actin	TGCCAGGGTACATTGTGGTA<br>TGCGTGACATCAAGGAGAAG	60	216	
LMEP	GCTAGCGCCTTGCCCGCGGCGGTTGCG<br>ACCGGTTTTTTTTTTTTTCTTTTCTTTG	60	3150	Overexpression
