# Supplementary material for: Revealing the Regulatory Mechanism of lncRNA-LMEP on Melanin Deposition Based on High-Throughput Sequencing in Xichuan Chicken Skin
Source: Genes (Basel). 2022 Nov 17;13(11):2143. doi: 10.3390/genes13112143 (PMC9690664; doi:10.3390/genes13112143)
Supplement: Supplementary file 1 [file genes-13-02143-s001.zip › Supplementary Table S2.pdf]

Table S2. Skin color characteristics of Xichuan black-bone chickens with YS and BS.

Group	Sex	Sample ID	Color Value	Analysis
YS	Female	YS1	55.88	RNA-seq and qRT-PCR
YS	Female	YS2	58.14	RNA-seq and qRT-PCR
YS	Female	YS3	57.05	RNA-seq and qRT-PCR
YS	Female	YS4	56.26	qRT-PCR
YS	Female	YS5	59.73	qRT-PCR
YS	Female	YS6	58.42	qRT-PCR
BS	Female	BS1	36.02	RNA-seq and qRT-PCR
BS	Female	BS2	37.83	RNA-seq and qRT-PCR
BS	Female	BS3	36.16	RNA-seq and qRT-PCR
BS	Female	BS4	38.96	qRT-PCR
BS	Female	BS5	35.35	qRT-PCR
BS	Female	BS6	35.42	qRT-PCR

Note: YS represents the yellow skin group, and BS represents the black skin group. The smaller the numerical grade of the black color of the skin, the greater the yellow coloration.
