# Supplementary material for: Revealing the Regulatory Mechanism of lncRNA-LMEP on Melanin Deposition Based on High-Throughput Sequencing in Xichuan Chicken Skin
Source: Genes (Basel). 2022 Nov 17;13(11):2143. doi: 10.3390/genes13112143 (PMC9690664; doi:10.3390/genes13112143)
Supplement: Supplementary file 1 [file genes-13-02143-s001.zip › Supplementary Table S3.pdf]

Table S3. Characteristics of the reads from the six chicken skin libraries

Sample ID	Total Reads	Clean Reads	Clean Bases	GC Content	%≥Q30	Mapped Reads	Unique Mapped Reads
YS-1	44,986,754	22,493,377	6,597,527,610	53.88%	86.44%	31,297,357 (69.57%)	29,280,025 (65.09%)
YS-2	45,862,450	22,931,225	6,701,085,310	54.56%	86.31%	31,819,922 (69.38%)	29,426,178 (64.16%)
YS-3	48,078,912	24,039,456	6,987,809,848	54.37%	86.14%	33,405,703 (69.48%)	30,410,773 (63.25%)
BS-1	44,092,374	22,046,187	6,429,548,622	54.23%	86.54%	31,006,065 (70.32%)	27,441,291 (62.24%)
BS-2	44,136,294	22,068,147	6,441,323,382	54.50%	86.07%	30,517,393 (69.14%)	28,149,679 (63.78%)
BS-3	48,775,554	24,387,777	7,148,900,028	55.14%	86.66%	34,422,875 (70.57%)	30,204,318 (61.93%)

Note: YS represents the yellow skin group, and BS represents the black skin group. Mapped Reads indicates the percent of mapped reads among all reads. Unique mapped Reads represents matched the reference genome in only one position.
