# Supplementary material for: Revealing the Regulatory Mechanism of lncRNA-LMEP on Melanin Deposition Based on High-Throughput Sequencing in Xichuan Chicken Skin
Source: Genes (Basel). 2022 Nov 17;13(11):2143. doi: 10.3390/genes13112143 (PMC9690664; doi:10.3390/genes13112143)
Supplement: Supplementary file 1 [file genes-13-02143-s001.zip › Supplementary Table S4.pdf]

Table S4. Coding potential of LMEP

Gene ID	Website	ORF Size	Ficket Score	Coding Probability	Coding Label
LMEP	CPAT	315	0.5032	0.0042003214976823	noncoding
	CPC	105	0.27991	0.0987826	noncoding
