# Supplementary material for: Revealing the Regulatory Mechanism of lncRNA-LMEP on Melanin Deposition Based on High-Throughput Sequencing in Xichuan Chicken Skin
Source: Genes (Basel). 2022 Nov 17;13(11):2143. doi: 10.3390/genes13112143 (PMC9690664; doi:10.3390/genes13112143)
Supplement: Supplementary file 1 [file genes-13-02143-s001.zip › Supplementary Table S5.pdf]

Table S5. lncLocator prediction result

Predicted location	Score
Cytoplasm	0.85506516
Nucleus	0.08972716
Ribosome	0.01153811
Cytosol	0.0344919
Exosome	0.00917767
