# Supplementary figures and images for: Revealing the Regulatory Mechanism of lncRNA-LMEP on Melanin Deposition Based on High-Throughput Sequencing in Xichuan Chicken Skin
Source: Genes (Basel). 2022 Nov 17;13(11):2143. doi: 10.3390/genes13112143 (PMC9690664; doi:10.3390/genes13112143)

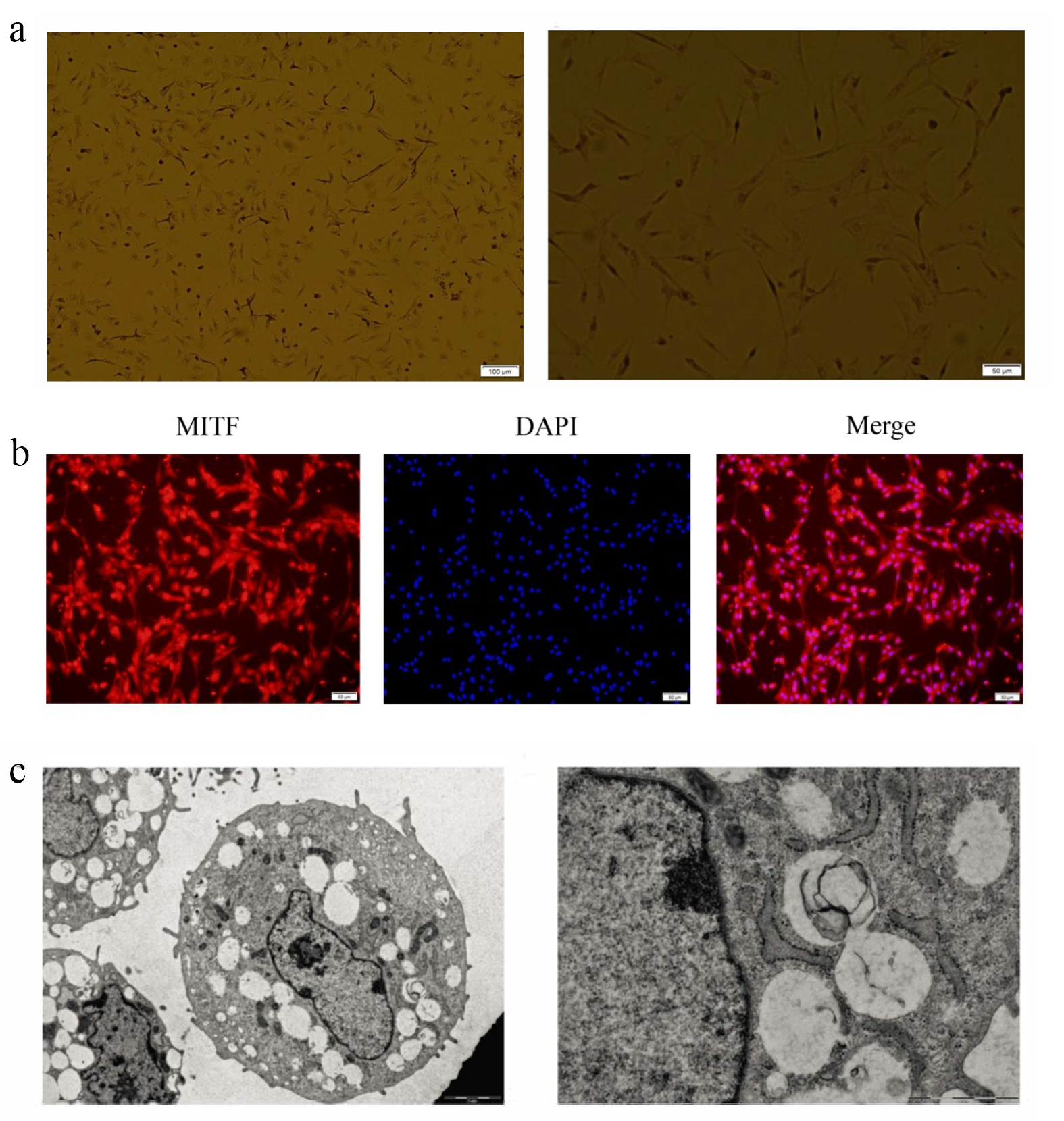

Supplement: Supplementary file 1 [file genes-13-02143-s001.zip › figure S1.tif]

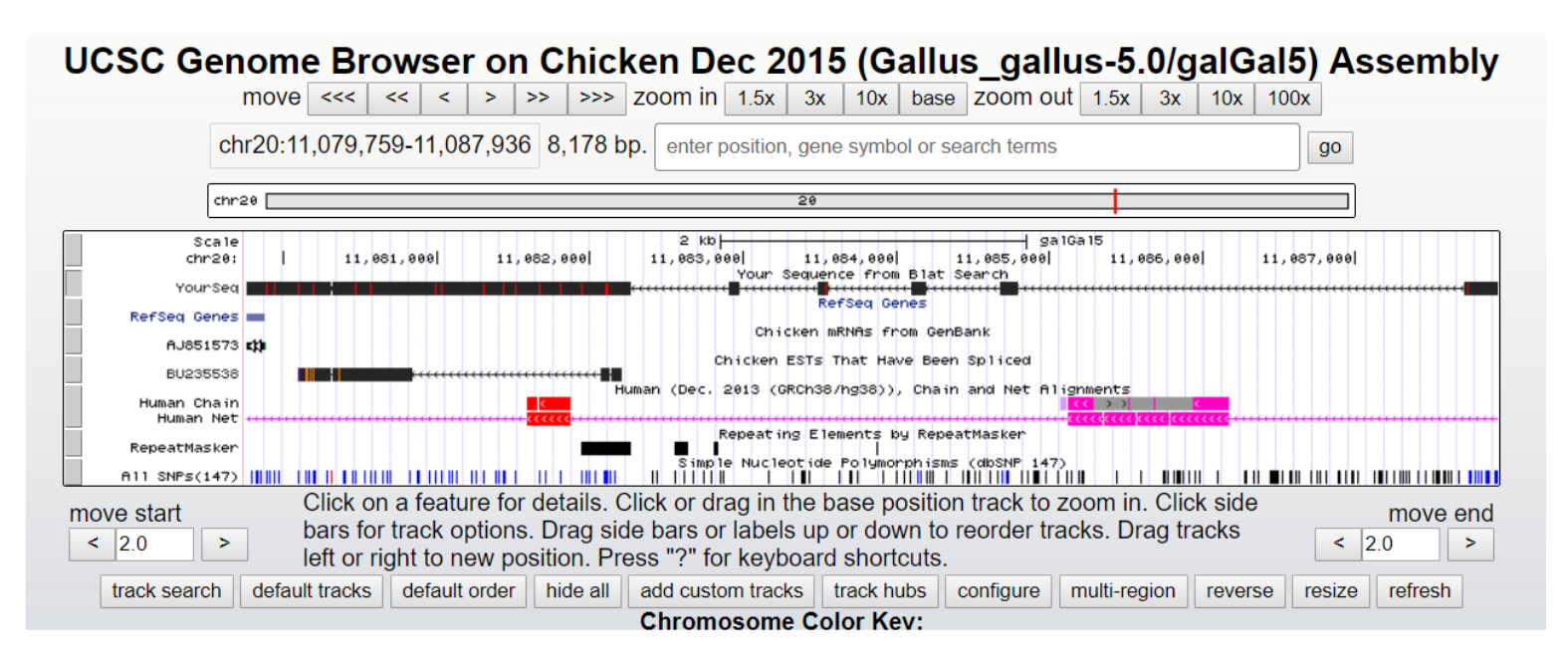

Supplement: Supplementary file 1 [file genes-13-02143-s001.zip › figure S2.tif]

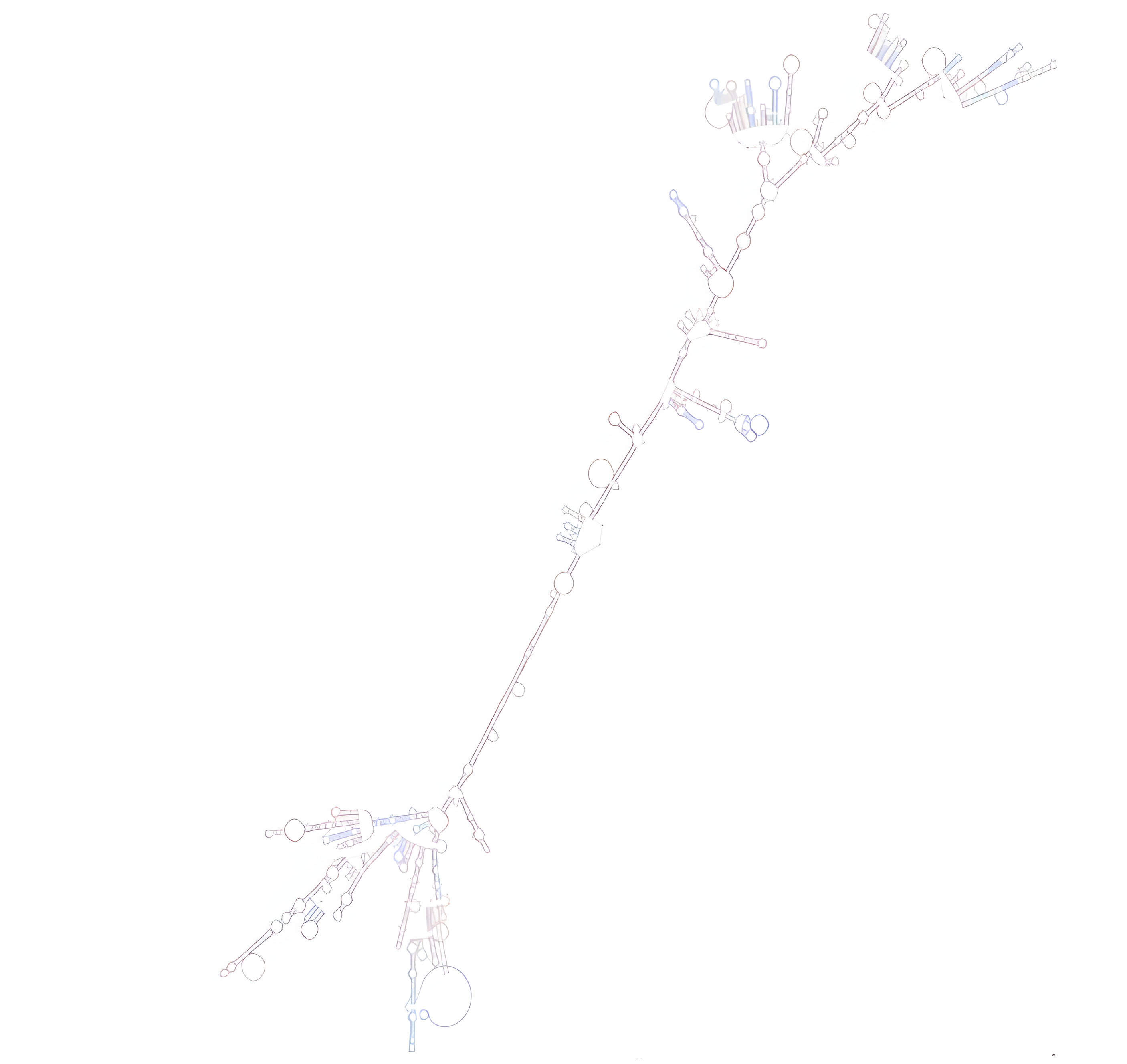

Supplement: Supplementary file 1 [file genes-13-02143-s001.zip › figure S3.png]
